# Imaging Diagnosis of Major Kidney and Urinary Tract Disorders in Children

**DOI:** 10.3390/medicina61040696

**Published:** 2025-04-10

**Authors:** Ahmad Aldughiem

**Affiliations:** Department of Nephrology, Wright State University, Dayton Children’s Hospital, One Children’s Plaza, Dayton, OH 45404, USA; aldughiema@childrensdayton.org; Tel.: +1-937-641-3304; Fax: +1-937-641-4600

**Keywords:** ultrasound (US), computed tomography (CT), magnetic resonance imaging (MRI), nuclear medicine, voiding cystourethrography (VCUG)

## Abstract

*Background and Objectives*: Diagnostic imaging is essential for evaluating urinary tract disorders, offering critical insights into renal pathology. This review examines the strengths, limitations, and clinical applications of various imaging modalities, with a focus on pediatric populations. *Materials and Methods*: A narrative review was conducted, synthesizing current literature on ultrasound (US), computed tomography (CT), magnetic resonance imaging (MRI), nuclear medicine, and voiding cystourethrography (VCUG). Relevant studies were selected based on diagnostic accuracy, clinical utility, and safety considerations. *Results*: US is the preferred first-line imaging due to its safety, accessibility, and cost-effectiveness. CT excels in detecting renal calculi, trauma, and malignancies but is limited by radiation exposure. MRI offers superior soft tissue contrast without radiation but is costly and often requires sedation. Nuclear medicine evaluates renal function and scarring, while VCUG remains the gold standard for diagnosing vesicoureteral reflux and posterior urethral valves. *Conclusions*: Imaging modalities are vital for diagnosing and managing urinary tract disorders, with selection based on clinical needs, patient age, and safety. Ultrasound is the primary choice for its non-invasiveness and cost-effectiveness, while CT, MRI, nuclear medicine, and VCUG provide essential structural and functional insights. A balanced approach ensures accuracy while minimizing patient risk, especially in pediatrics.

## 1. Introduction

The evaluation of pediatric renal and urinary tract disorders is crucial for accurate diagnosis and management, ranging from congenital anomalies to acquired diseases. Pediatric patients present unique challenges due to anatomical, physiological, and developmental differences, requiring specialized imaging techniques for assessing kidney and urinary tract structure and function [1,2].

Diagnostic imaging is vital in pediatric nephrology and urology, offering structural information, evaluating renal function, detecting anomalies, monitoring progression, and guiding interventions. Common modalities include US, VCUG, CT, MRI, and nuclear medicine [2,3,4].

US is the first-line modality due to its safety, non-invasiveness, and ability to detect structural abnormalities [5,6,7]. For more complex cases or when additional functional data is required, CT, MRI, and nuclear medicine techniques such as renal scintigraphy are used, though they may involve radiation exposure or sedation [4,5,8].

This review examines these imaging modalities, highlighting their indications, advantages, limitations, and the evolving role of advanced techniques. Physicians should be aware of the benefits, risks, and selection criteria for each imaging method [1,2].

## 2. Methodology

### 2.1. Study Design

This narrative review synthesizes current pediatric renal imaging techniques, emphasizing diagnostic accuracy, clinical applications, and safety. The included studies, detailed in the Literature Matrix (Table 1), provide a comprehensive overview of various imaging modalities.

### 2.2. Data Collection and Selection

A systematic search was conducted using PubMed and Google Scholar (1980–2024) with keywords related to pediatric renal imaging. Sources included peer-reviewed articles, textbooks, systematic reviews, and meta-analyses. Studies were selected based on credibility, relevance, and methodological rigor.

A structured selection process was followed:

Identification: Studies were retrieved, and duplicates removed.

Screening: Titles and abstracts were reviewed for relevance.

Eligibility: Full-text studies were assessed based on inclusion/exclusion criteria.

Inclusion: Final selection focused on imaging modalities’ clinical application, accuracy, and safety.

### 2.3. Inclusion and Exclusion Criteria

Included: Studies on diagnostic accuracy, clinical applications, and imaging safety.

Excluded: Studies with insufficient data, small sample sizes (unless unique), or case reports.

### 2.4. Data Categorization and Analysis

Imaging modalities were categorized as US, VCUG, CT, MRI, and Nuclear Medicine. Each was evaluated for technical aspects, clinical utility, and comparative advantages (e.g., radiation exposure, sensitivity, specificity).

### 2.5. Ethical Considerations

All images and figures presented in this article were obtained from patients’ charts, ensuring compliance with ethical guidelines and confidentiality standards. Patient privacy was maintained throughout the review process, with no sensitive information disclosed.

## 3. Ultrasonography

Ultrasound (US) is essential for evaluating the urinary tract, particularly for assessing kidney shape, size, and hydronephrosis. It is widely available, cost-effective, safe, and non-invasive, making it ideal for frequent renal assessments, including pre- and post-procedural evaluations like stent or catheter placement and kidney stone screening. However, image quality depends on operator skill and may be limited in obese or uncooperative patients [1,2,4,5,6].

### 3.1. Procedure

US requires minimal preparation and is typically performed with the patient in a supine position. The kidneys are scanned in longitudinal and transverse planes with a transducer at the flanks. Adjusting the patient’s position can enhance visualization, and a full bladder is preferred for optimal assessment.

High-frequency ultrasound provides superior resolution, particularly in infants and young children, though image quality may be reduced in obese patients.

A comprehensive US study includes:

- Renal shape, size, cortical thickness, and echogenicity
- Corticomedullary differentiation
- Renal pelvis, ureters, and bladder

Ureters are usually not visible unless dilated. Kidney length is best measured in the supine or contralateral position. Normative kidney size data for different age groups and reference values for kidney and bladder volumes are available (Table 2) [9,10,16,17,18,19,20].

Kidney length measurements can vary based on examiner expertise and observer variability, making size alone an unreliable indicator of kidney growth over short periods [21]. In over 50% of children, the left kidney is slightly longer than the right, though the right kidney may sometimes be longer or of equal length [22].

The ultrasound appearance of kidneys changes with age. Neonates and infants have a higher proportion of medullary tissue compared to cortical tissue, and neonates exhibit increased cortical echogenicity, which decreases after birth [18,23]. Nonrenal conditions can also cause transient cortical echogenicity [19,24].

**Figure 1:**
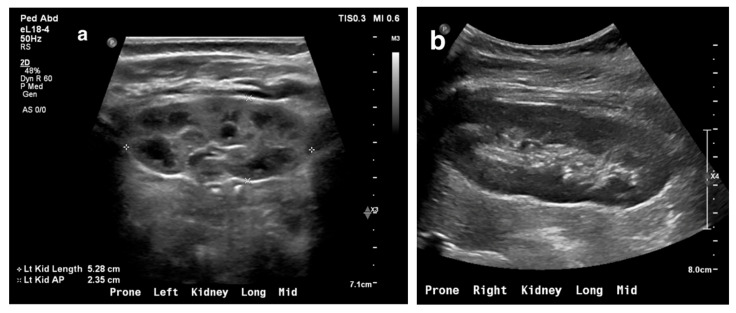
(**a**) Normal ultrasound of a 1-month-old infant’s kidney, showing prominent hypoechoic medullary pyramids. (**b**) Normal renal ultrasound of a 4-year-old child.

Corticomedullary differentiation is well-defined in term newborns, with hypoechoic and prominent medullary pyramids (Figure 1a) [23]. By one year of age, medullary echogenicity increases, and the pyramids become less prominent (Figure 1b) [18].

**Figure 2:**
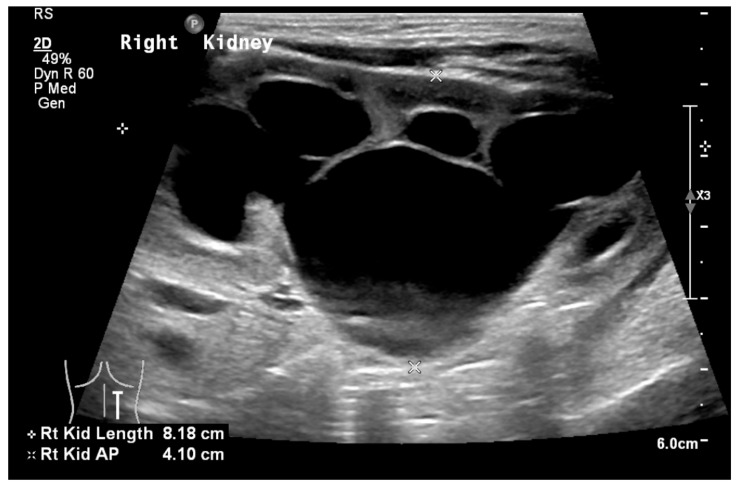
Renal ultrasound (US) showing hydronephrosis in a 5-day-old male infant with ureteropelvic junction obstruction.

US is highly effective for evaluating and monitoring hydronephrosis. Hydronephrosis may result from urinary obstruction or vesicoureteral reflux (VUR) (Figure 2). In some cases, mild hydronephrosis can be a normal variant.

Various systems are used to classify hydronephrosis, including the Urinary Tract Dilatation (UTD) and Society for Fetal Urology grading systems (Table 3) [12,25].

### 3.2. US Applications

US is valuable for assessing bladder and wall thickness, detecting ureteroceles and masses [26], and evaluating renal vascular flow using color and power Doppler. This helps identify conditions like renal artery disease, renal vein thrombosis, and arteriovenous fistulas [27,28].

US is essential for diagnosing and managing various kidney conditions, including chronic kidney disease, acute kidney injury, hematuria, nephrolithiasis, cystic kidney disease, kidney transplant dysfunction, abdominal masses, and monitoring abnormal fetal ultrasounds. It also guides invasive procedures such as real-time localization of native and transplanted kidneys, peritoneal catheter insertion, diagnosing tunnel infections in peritoneal dialysis, assessing intravascular volume via the inferior vena cava, and detecting lung water [6].

### 3.3. Limitations

US is operator-dependent, less effective in obese patients, and has lower sensitivity for small renal calculi [2,4,11].

## 4. Emerging Ultrasound Technologies

### 4.1. Three-Dimensional Ultrasound (3D US)

Three-dimensional (3D) US provides high-resolution images with excellent spatial and contrast resolution, enabling accurate anatomical reconstruction, better tissue differentiation (e.g., fluid-filled structures), and improved identification of renal stones and lesions [7]. It allows volumetric evaluation of the kidney, offering comparable results to nuclear medicine and MRU, reducing the need for these procedures [29]. Three-dimensional (3D) US also enhances the visualization of the collecting system, potentially decreasing the need for fluoroscopy or MRU [2,7].

### 4.2. Contrast-Enhanced Ultrasound (CEUS)

CEUS uses microbubble contrast agents to enhance blood flow and tissue vascularity visualization. These agents improve ultrasound signal clarity, enabling real-time imaging of blood flow dynamics and offering greater sensitivity in detecting perfusion abnormalities compared to Doppler imaging [30].

### 4.3. Contrast-Enhanced Voiding Urosonography (CEVUS)

CEVUS is used to detect vesicoureteral reflux (VUR) by instilling an ultrasound contrast agent intravesically. It also visualizes the urethra, making it suitable for assessing the entire urinary tract. The procedure involves inserting a catheter into the bladder, followed by contrast administration and imaging during filling and voiding cycles. Two or three voiding cycles are typically performed [31].

#### 4.3.1. Indications

CEVUS is useful for evaluating VUR, hydronephrosis, recurrent UTIs, and urethral anomalies [2,31].

#### 4.3.2. Advantages

The main advantage of CEVUS is the elimination of radiation exposure, although bladder catheterization is still required [31].

#### 4.3.3. Limitations

CEVUS shares the limitations of other invasive procedures, such as difficulties in patients with severe scoliosis or crossed-fused renal ectopia. Additionally, it may be challenging to obtain a single image of the entire urinary tract, complicating interpretation [31].

## 5. Computed Tomography (CT)

**Figure 3:**
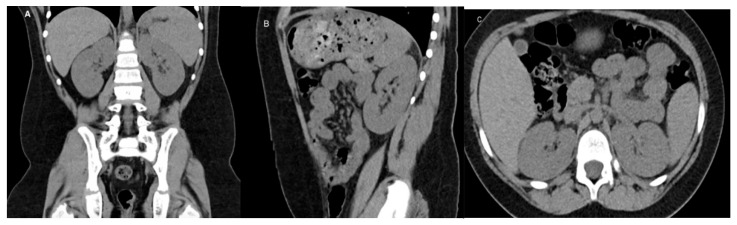
CT of a normal 8-year-old boy: (**A**) Coronal image, (**B**) Sagittal image, (**C**) Axial image.

CT is a cross-sectional imaging technique widely used for diagnosing renal diseases in children. It provides high-resolution images of the urinary tract and retroperitoneal structures (Figure 3). However, radiation exposure is a key concern, and to minimize risk, it is recommended to limit the field of view and use weight-based exposure parameters [32,33].

### 5.1. Technique

Multidetector CT scanners quickly obtain thin-section data, which can be reconstructed into sagittal, coronal, and oblique planes to display the kidneys, ureters, bladder, and surrounding structures. IV contrast is typically used, except when evaluating urinary calculi. However, contrast agents can cause nephrotoxicity, especially in cases of acute kidney injury (AKI), chronic kidney disease (CKD), dehydration, or when nephrotoxic drugs are involved [34,35]. To reduce contrast nephrotoxicity, it is recommended to use low-osmolality nonionic agents, ensure adequate hydration, and administer the minimum contrast dose needed.

### 5.2. Application

CT is the most sensitive modality for detecting urinary calculi without contrast, but US is often used for surveillance to minimize radiation exposure [11]. CT is also effective for the rapid evaluation and staging of renal tumors and assessing urinary tract trauma. Although CT provides high-resolution images of renal scarring, ^99m^Tc-DMSA is more commonly used for this purpose. Renal artery stenosis can be evaluated through CT angiography [36].

### 5.3. Limitations

Radiation exposure limits its use in pediatrics, and contrast agents carry a risk of nephrotoxicity [2,4,5].

## 6. Voiding Cystourethrography (VCUG)

Voiding cystourethrography (VCUG) is the gold standard for detecting vesicoureteral reflux (VUR) and provides valuable information about the bladder and urethra [14]. It is commonly indicated for recurrent urinary tract infections and is often found in cases of hydronephrosis and hydroureter.

### 6.1. Procedure

A VCUG is conducted using fluoroscopy. While no specific preparation is required, it is crucial to explain the procedure to both the parents and the child, if age-appropriate. The presence of parents and, in some cases, a child-life specialist can help ease anxiety during the study. However, if the patient is extremely anxious and uncooperative, oral sedation with midazolam may be used, offering the added benefit of its amnestic effect [37].

An initial abdominal radiograph is taken before filling the bladder to check for calculi or bony abnormalities. The stool burden may also be assessed. Next, the bladder is sterilely catheterized with a feeding tube, and a urine specimen is typically obtained for urinalysis and culture. A contrast material is instilled into the bladder under gravity, and an early filling film is taken to identify any filling defects, such as ureteroceles or bladder tumors. Images are captured before and after voiding, and multiple cycles of bladder filling and voiding can enhance the sensitivity of VCUG in detecting VUR [4,5].

### 6.2. Application

**Figure 4:**
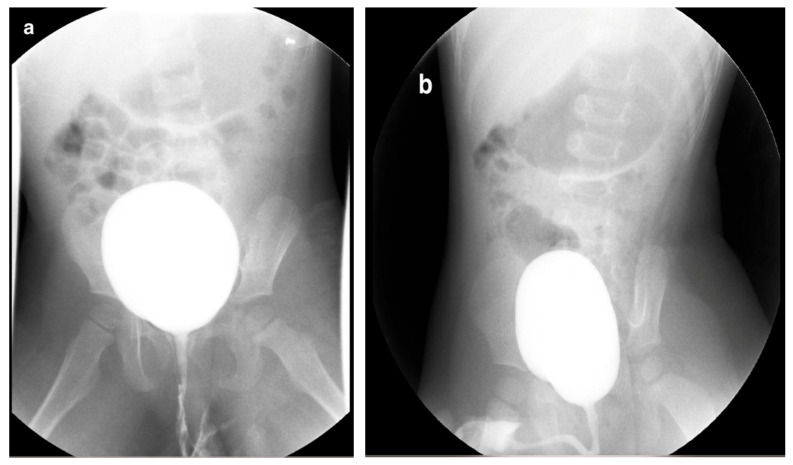
Voiding cystourethrogram. (**a**) Normal voiding image of a 2-year-old female. (**b**) Normal voiding image of a 2-year-old male.

VCUG is the gold standard for diagnosing and grading VUR, offering a comprehensive assessment of the bladder and urethra. VUR is generally suspected when there is urinary tract dilation (UTD), ureteral dilation, or abnormal ultrasound findings, such as uroepithelial thickening or scarring, especially following a first febrile urinary tract infection (UTI). Other indicators include recurrent UTIs, dysfunctional voiding (e.g., neurogenic bladder dysfunction), or bladder outlet obstruction [2]. The grading of VUR is based on the International Reflux Study in Children [38]. Capturing images during voiding is essential for evaluating the urethra and identifying posterior urethral valve (PUV) in male children. A normal urinary bladder appears as a smooth-walled, globular structure, while the urethral walls are smooth, and the posterior urethra is observed as a nondistended, smooth-walled passage (Figure 4). In specific cases, especially in children with trauma or dysuria, a retrograde urethrogram—dedicated exclusively to imaging the urethra—may be preferred over a VCUG [2].

### 6.3. Complications

The most common complication of VCUG is radiation exposure. Other risks include iatrogenic infection, chemical cystitis, and urethral trauma. Catheterization can be distressing for some families, and sedation with midazolam may be necessary for extremely anxious children [39].

### 6.4. Limitations

VCUG is invasive, requiring catheterization, and involves radiation exposure.

## 7. Intravenous Pyelography (IVP)

IVP use has decreased with the advent of alternative imaging methods such as ultrasound (US), CT, and MRI. The main side effects of IVP are radiation and contrast exposure [2,4,5].

### 7.1. Technique

IV access is required for contrast injection. An initial film is taken in the supine position before injection. Images of the kidneys are captured 5–7 min after contrast to show nephrograms and calyces, with additional images taken at 15 and 30 min. Delayed images may provide more information in cases of urinary obstruction.

### 7.2. Indications

**Figure 5:**
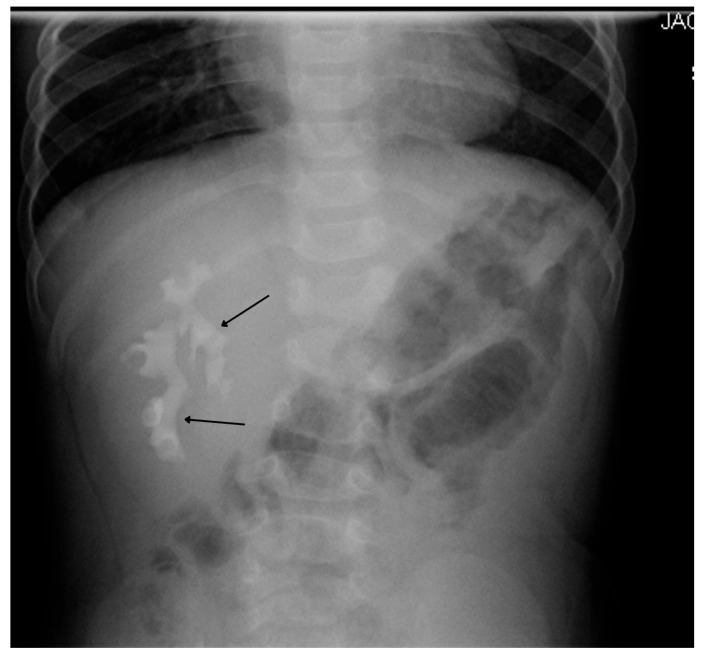
IVP shows right crossed renal ectopia (indicated by arrows).

IVP is useful for evaluating ureteroceles, renal scarring, ectopic ureters, and obstructive disease (Figure 5), but has been largely replaced by US, CT, MRI, and renal scanning.

## 8. Nuclear Medicine

Nuclear medicine uses radiotracers that emit photons detected by a scintillation detector. It provides quantitative functional data, offering advantages over other imaging techniques. Scintigraphy and radionuclide cystography are commonly used, with pharmacologic agents like furosemide and captopril enhancing diagnostic accuracy. Nuclear medicine typically uses less radiation than procedures like VCUG, IVP, or CT [1,2].

### 8.1. Procedure

#### 8.1.1. Renal Cortical Imaging

^99m^Tc-DMSA (dimercaptosuccinic acid) is the preferred radiotracer for renal cortical imaging, with about 60% of it being absorbed by cells in the proximal tubule. Imaging is conducted 1.5 to 3 h after injection to allow for gradual accumulation in the kidneys. Left and right posterior oblique images are captured to improve diagnostic accuracy.

##### Application

Renal cortical scans diagnose renal scarring and acute pyelonephritis in patients with febrile UTIs. Single-photon emission computed tomography (SPECT) is an alternative that provides greater sensitivity in detecting renal scarring [40,41].

1. Acute Pyelonephritis

**Figure 6:**
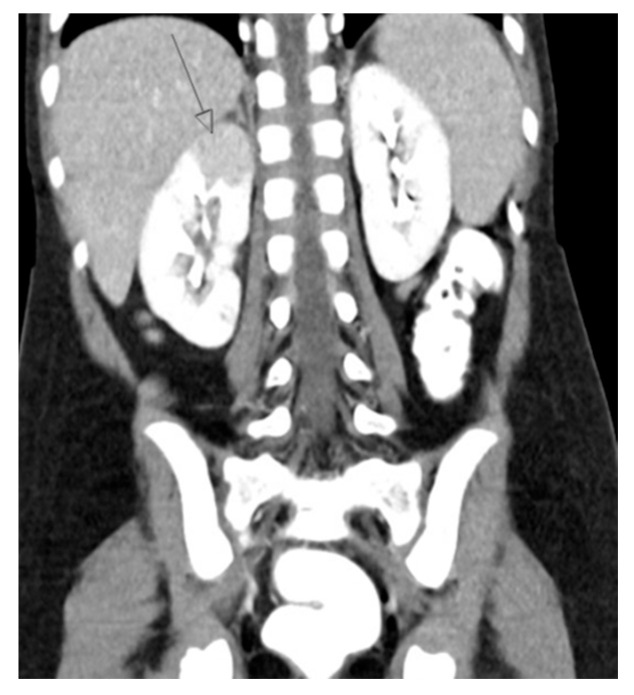
CT shows right sided pyelonephritis (indicated by the arrow) in a 4-year-old female.

DMSA scanning is the gold standard for diagnosing acute pyelonephritis, as clinical criteria alone are not always reliable [42,43]. While US may show renal enlargement and poor corticomedullary differentiation, it is less sensitive and specific compared to other imaging modalities. MRI and CT offer good sensitivity (Figure 6), but DMSA remains preferred.

In acute pyelonephritis, reduced radiotracer uptake without volume loss of renal tissue is observed. Photogenic defects can be focal, multifocal, or diffuse, with focal defects being the most common. These defects are not specific to acute pyelonephritis and may also be seen in cysts, infarcts, masses, or traumatic lesions, requiring alternative imaging for clarification and clinical context interpretation [8].

2. Renal Scarring

**Figure 7:**
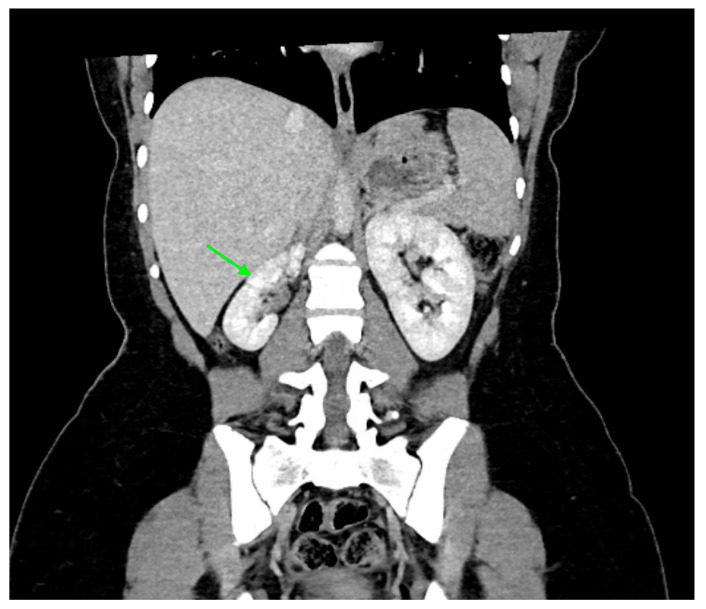
A CT scan of a 15-year-old female with a history of recurrent pyelonephritis shows a scarred and atrophic right kidney (indicated by green arrow).

Acute pyelonephritis may cause renal scarring, potentially altering kidney size and function (Figure 7). On DMSA scans, renal scars appear as photogenic defects associated with renal parenchymal volume loss. If pyelonephritis is suspected on a DMSA scan, it is recommended to repeat the study after 6–12 months to assess for scarring, as most acute pyelonephritis foci resolve without scar formation [44].

#### 8.1.2. Dynamic Renal Scintigraphy

For renal functional imaging, ^99m^Tc-DTPA and ^99m^Tc-MAG3 are the primary radiotracers.

^99m^Tc-DTPA is excreted via glomerular filtration and is not reabsorbed or secreted by the tubules.

^99m^Tc-MAG3 is cleared primarily by tubular secretion, making it more useful for evaluating the collecting system and generally superior to ^99m^Tc-DTPA due to faster excretion.

Furosemide is used with ^99m^Tc-MAG3 to assess urinary obstruction. After the radiotracer injection and water intake, a Foley catheter is placed to reduce intravesical pressure. Dynamic renal scans are performed for 30 min, followed by furosemide administration. Sequential images are taken for another 30 min. A non-obstructed kidney drains quickly, while an obstructed kidney drains slowly [45,46].

#### 8.1.3. Radionuclide Cystography (RNC)

RNC is used to diagnose vesicoureteral reflux (VUR). Similar to VCUG, a catheter is inserted into the bladder, and a radioisotope with saline is infused. Imaging starts at the beginning of filling and continues through voiding, enabling reflux detection at any point during the exam.

RNC offers several advantages over VCUG, including significantly lower radiation exposure and greater sensitivity in detecting VUR due to continuous monitoring. However, VCUG remains more useful for evaluating the bladder and urethra [2,15].

## 9. Magnetic Resonance Imaging (MRI)

- MRI provides high-resolution images of the urinary tract without radiation risk. It can image the abdomen in multiple planes, though sedation is often required for infants and younger children.
- While MRI contrast agents are less nephrotoxic than iodinated contrasts, gadolinium-based contrast agents (GBCAs) can cause nephrogenic systemic fibrosis (NSF), a rare but serious condition, particularly in patients with advanced chronic kidney disease (CKD, GFR < 30 mL/min/1.73 m^2^). The need for GBCAs should be carefully considered in patients with CKD or acute kidney injury (AKI) [47,48].

### 9.1. Application

Magnetic resonance urography (MRU) provides detailed information about the urinary tract’s anatomy and function, including split renal function and GFR estimation. It is especially useful for evaluating congenital anomalies, cystic and solid renal masses, renal vascular abnormalities, and hydronephrosis. MRU can also assess urinary obstruction based on the response to fluid and diuretic administration [2,13,49].

### 9.2. Limitations

- Expensive, requires sedation in children.
- Longer scan times compared to CT.

## 10. Fetal Urogenital Imaging

Congenital urinary tract (UT) abnormalities are detected prenatally in 3–4% of pregnancies [50]. Fetal ultrasound (US) is crucial for assessing amniotic fluid volume, identifying UT anomalies, and detecting associated conditions like VACTREL syndrome. Fetal kidneys become visible on US by the 12th–13th week and appear lobulated with hypoechoic pyramidal structures [51]. Hydronephrosis, the most common renal abnormality, often resolves before birth, with only 20% persisting post-delivery. Fetal hydronephrosis is measured by the anteroposterior pelvic diameter (APD), which should be under 3 mm, though it varies with gestational age, bladder fullness, and maternal hydration [51,52].

Antenatal hydronephrosis is graded using the Society of Fetal Urology (SFU) system, with the severity often predicting postnatal outcomes [51,53]. US can assess calyceal and ureteral dilation and renal parenchymal thinning, which, when bilateral, increases the risk of oligohydramnios. Renal US is typically avoided in the first days of life to prevent false negatives due to low urine output, with follow-up recommended at least 4 weeks after birth unless immediate intervention is required [54].

Fetal US can also detect conditions like multicystic dysplastic kidneys (MCDK), polycystic kidneys, posterior urethral valves (PUV), vesicoureteral reflux (VUR), duplex collecting systems, and a single kidney [55]. Fetal MRI plays a key role in characterizing complex urogenital abnormalities, including PUV, junctional parenchymal junction (JPJ) obstructions, and conditions like bladder exstrophy and cloacal malformations [56].

## 11. Dysplastic Kidney

**Figure 8:**
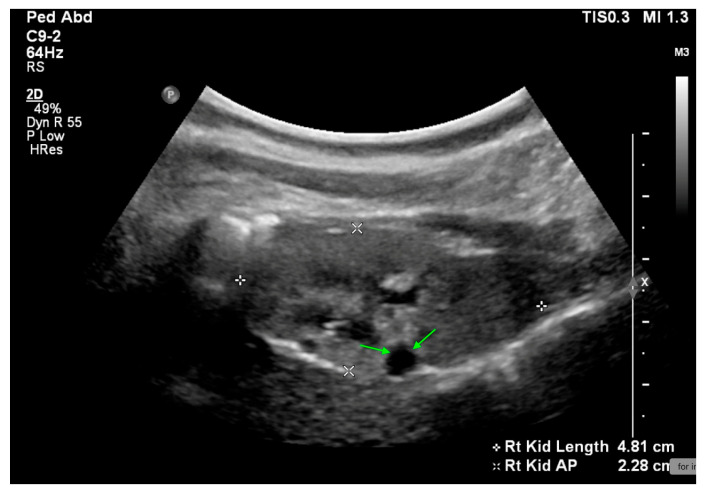
US reveals a small echogenic right kidney with a renal cyst (highlighted by green arrows) in a 2-year-old female.

Renal dysplasia is characterized by the presence of primitive ducts and cartilage. It is primarily diagnosed histologically, though US can reveal increased echogenicity and reduced kidney size, US may also show cysts of varying sizes and numbers (Figure 8).

## 12. Multicystic Dysplastic Kidney (MCDK)

**Figure 9:**
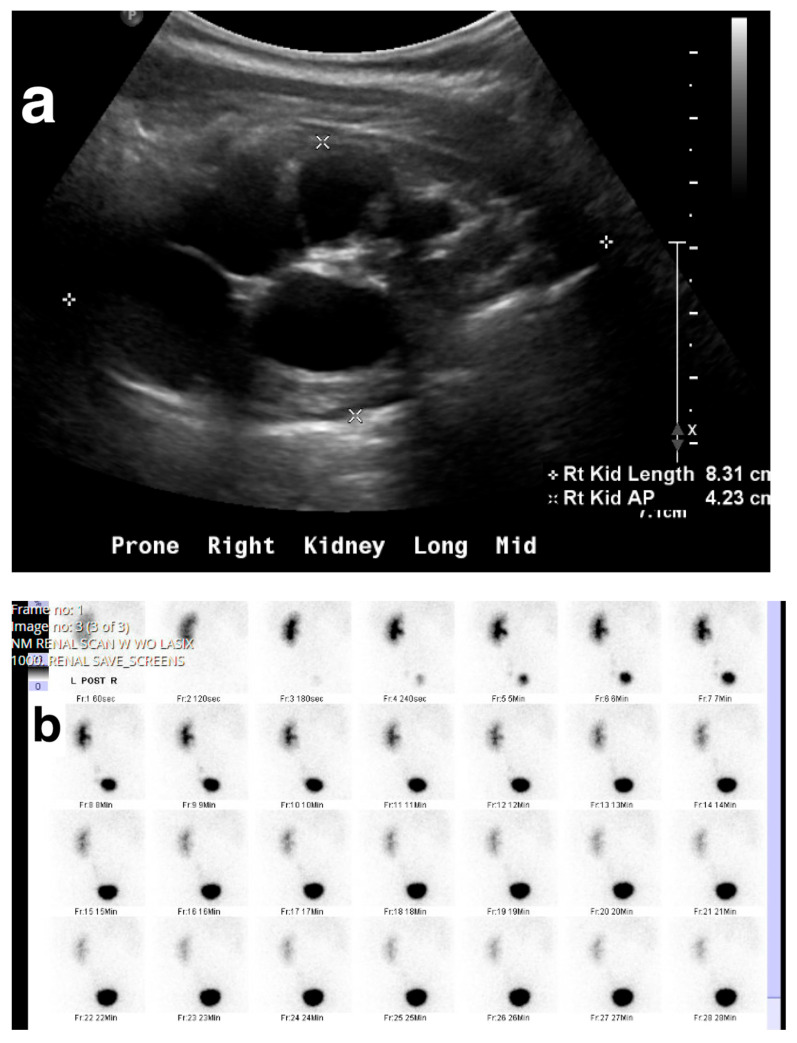
(**a**) US shows right MCDK in a 23-month-old male. (**b**) Renal scan shows no uptake in the right kidney.

MCDK is a common prenatal anomaly, occurring in about 1 in 2500 newborns [57]. On US, MCDK appears as multiple non-communicating cysts of different sizes surrounded by abnormal parenchyma (Figure 9). Differentiating MCDK from severe UPJ obstruction can be challenging. In MCDK, the cysts are non-communicating, while in hydronephrosis, the calyces extend outward from the dilated pelvis, surrounded by functional renal tissue [58].

To clarify the diagnosis, additional imaging such as MAG-3 scintigraphy can confirm the absence of tracer uptake in MCDK, while tracer uptake will be seen in hydronephrosis. VUR and other renal anomalies can be present in the contralateral kidney in up to 25% of cases [59], so a VCUG may be necessary to rule out VUR in MCDK patients, especially if they have UTIs.

Cyst involution in MCDK is common over time, with many cases showing improvement or resolution in the following years [59,60].

## 13. Obstructive Uropathy

**Figure 10:**
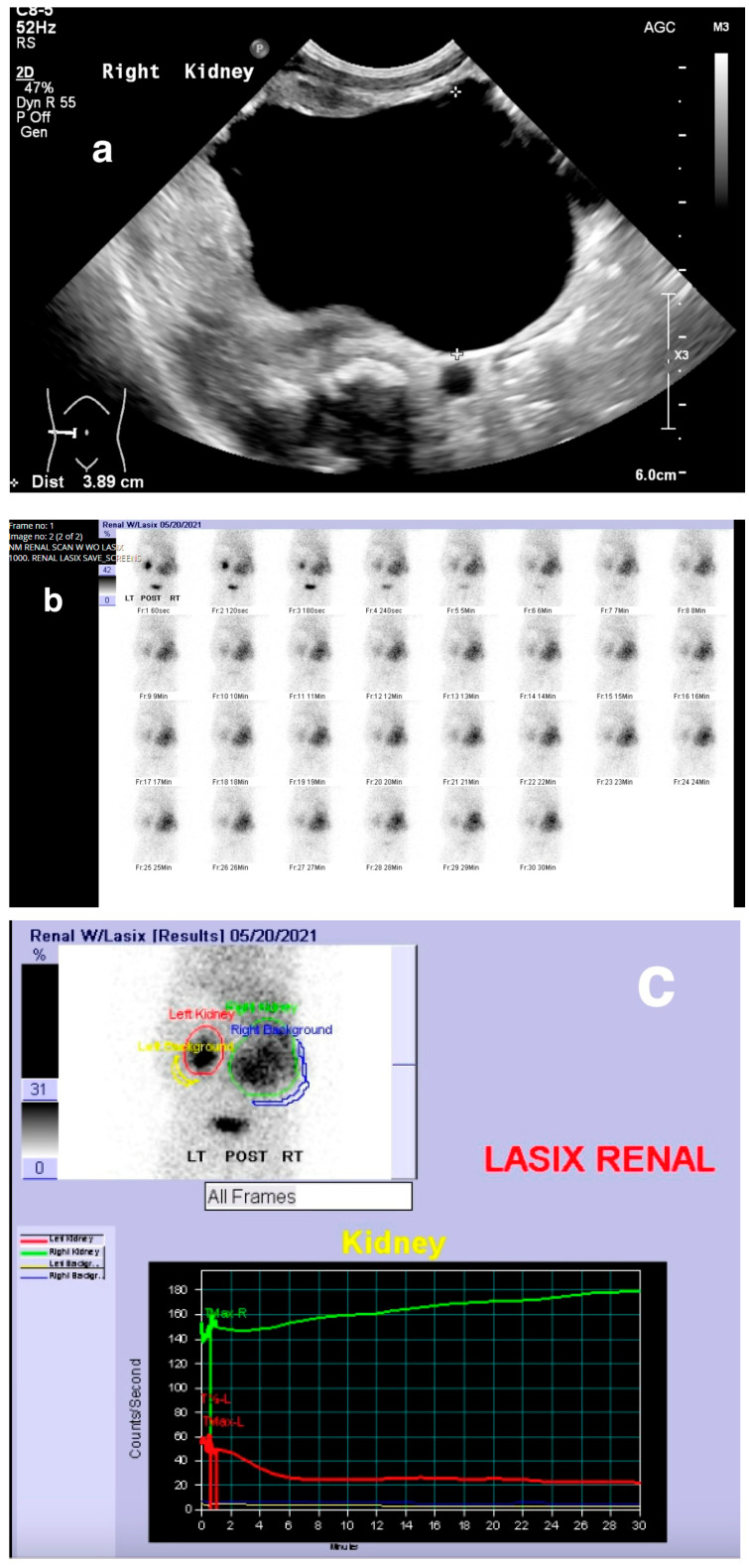
Right ureteropelvic junction obstruction in a neonate. (**a**) US shows severe dilatation of the right renal pelvis. (**b**) Nuclear medicine shows retention of radiotracer in the right kidney. (**c**) Mild drainage occurs in the right kidney after Lasix, with a T ½ exceeding 30 min.

**Figure 11:**
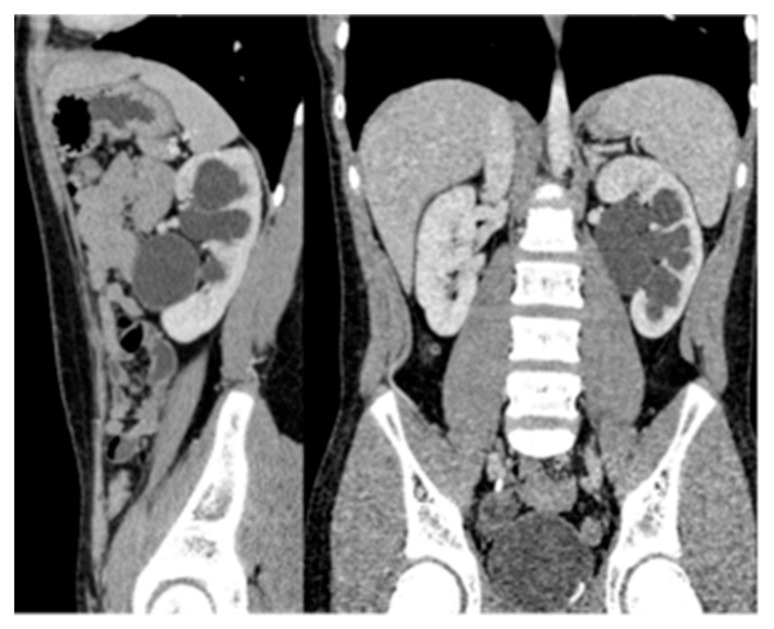
CT scan showing left ureteropelvic junction obstruction.

Obstruction at the ureteropelvic junction (UPJ) is a common congenital urinary tract obstruction. It is often diagnosed via antenatal ultrasound (US), which can reveal significant pelvic and calyceal dilation. Postnatally, UPJ obstruction can be detected using US, CT, or MRI, but dynamic nuclear medicine remains the gold standard for diagnosis (Figure 10 and Figure 11).

**Figure 12:**
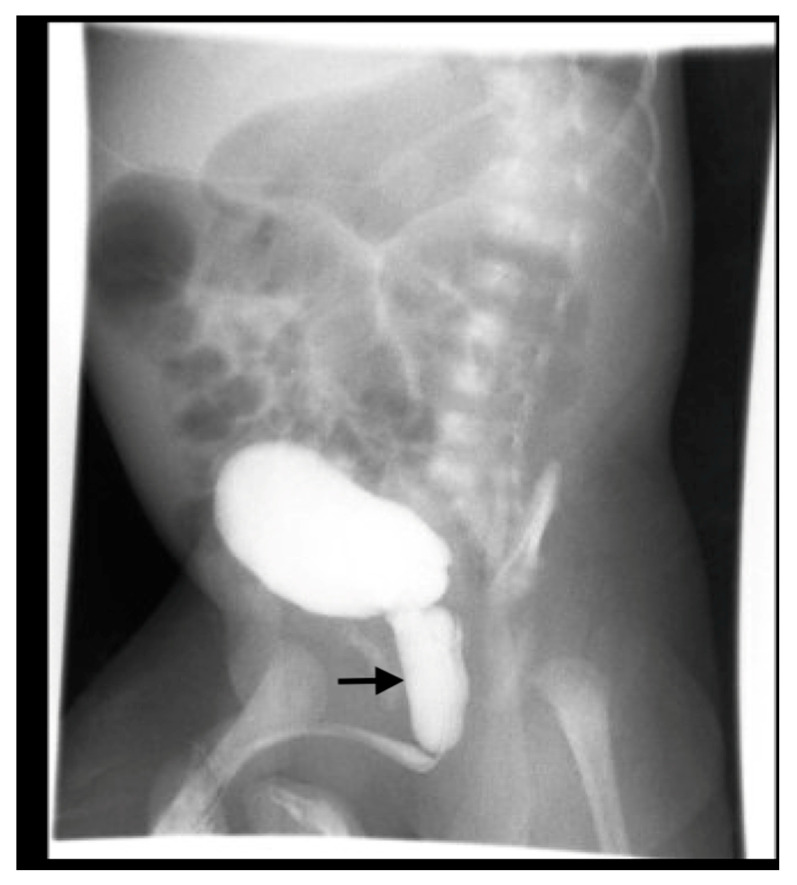
Voiding cystourethrogram shows dilatation of the posterior urethra (arrow) in a 4-week-old boy with PUV.

Bilateral hydronephrosis may also result from lower urinary tract obstruction, such as PUV. Fetal US can show a thick-walled, trabeculated bladder and a dilated prostatic urethra, which forms the characteristic “keyhole sign” in PUV. VCUG is the gold standard for diagnosing PUV (Figure 12).

## 14. Duplex Collecting System

**Figure 13:**
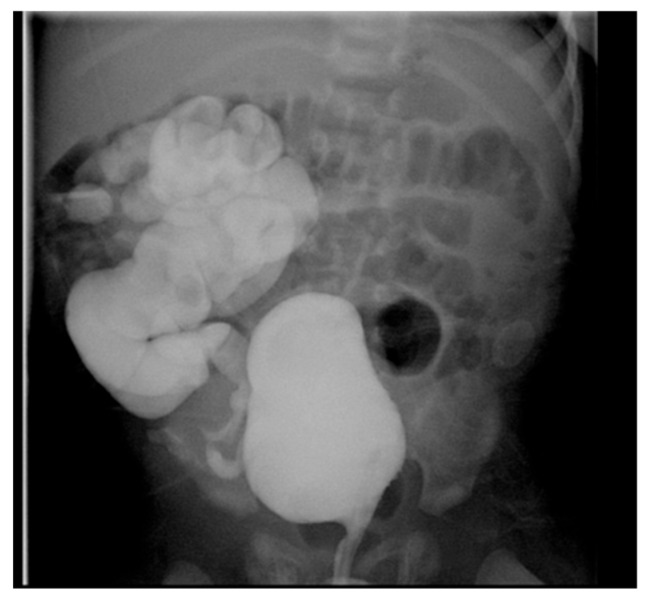
VCUG shows Grade 4–5 VUR into the lower pole of the duplex system. VUR is superimposed on residual contrast in the upper pole.

A duplex collecting system is a common urinary anomaly, but it is clinically significant only when associated with urinary obstruction. It may also be linked to other anomalies such as ectopic ureters, VUR, ureteroceles, and UPJ obstruction. The lower pole is typically associated with VUR, while the upper pole often involves obstruction at the distal ureter (Figure 13).

US can reveal the division of the kidney but usually cannot visualize the bifid ureters. MRU is particularly useful for detailing ureteral duplication and characterizing distal ureters, which is essential for surgical planning [61].

## 15. Horseshoe and Ectopic Kidneys

**Figure 14:**
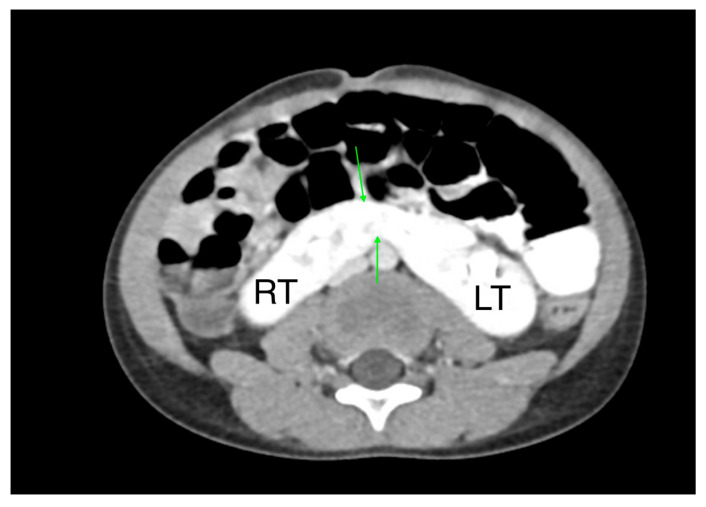
A CT scan reveals a horseshoe kidney, with fusion of the lower poles indicated by green arrows.

- A horseshoe kidney is the most common renal fusion anomaly, occurring in about 1 in 400 live births. In this condition, both kidneys are fused at their lower poles, forming a “horseshoe” shape (Figure 14)
- Renal ectopia occurs in approximately 1 in 900 births and is typically unilateral. In about 40% of cases, the ectopic kidney is located in the pelvis. Crossed ectopia occurs when one kidney crosses the midline and fuses with the other, often resulting in a “crossed-fused” appearance.

US is typically diagnostic for these conditions, as it can identify the location and structure of the kidneys. However, additional imaging, such as intravenous pyelography (IVP), CT, MRI, or renal scintigraphy, may be needed for further confirmation and to assess any associated abnormalities or complications (Figure 5).

**Figure 15:**
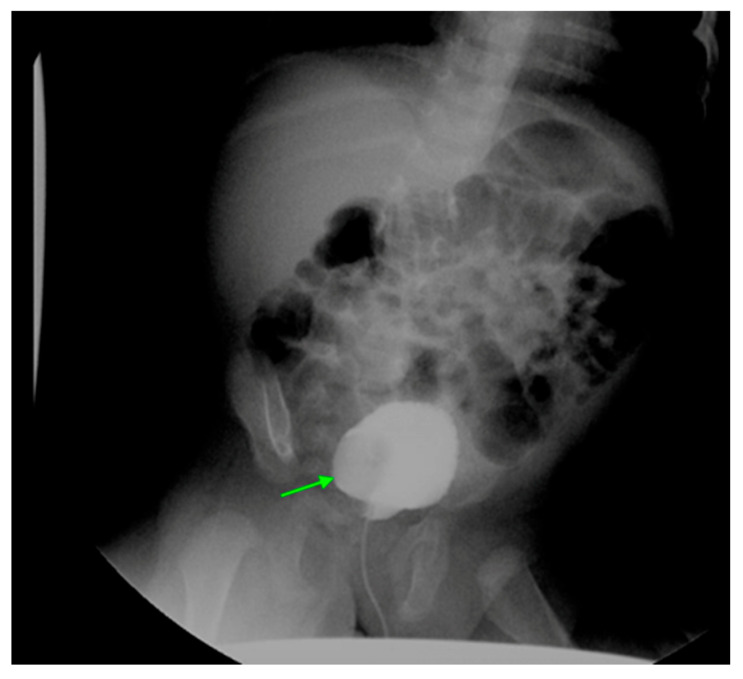
VCUG reveals a ureterocele on the right side of the bladder, identified as a filling defect marked by the green arrow.

Ureteroceles are abnormal expansions of the distal ureter within the bladder wall and are often linked to duplex collecting systems. They may obstruct the upper pole and urethra during urination. Imaging techniques such as US, VCUG, IVP, and MRU are useful for diagnosing ureteroceles. On VCUG, a ureterocele appears as a filling defect in the bladder during the early bladder-filling phase (Figure 15) [62].

## 16. Vesicoureteral Reflux

**Figure 16:**
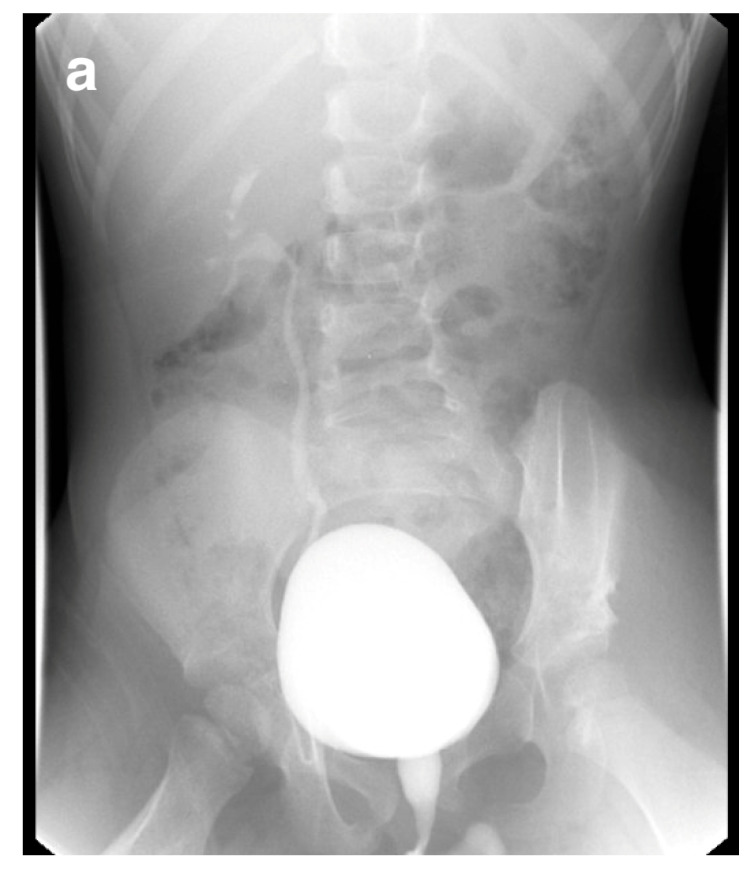

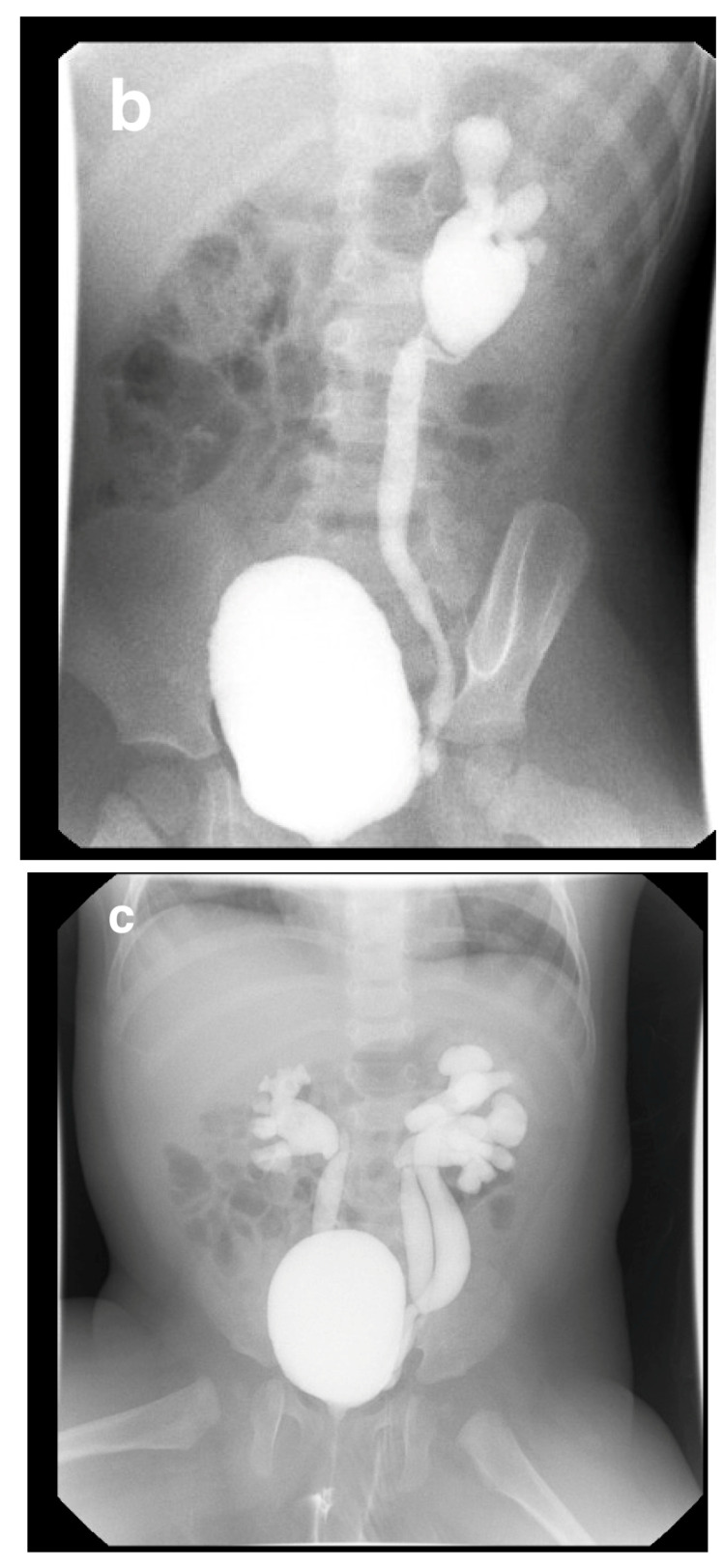
VCUG shows: (**a**) right VUR grade II in a 4-year-old female, (**b**) left VUR grade III in a 4-year-old female, and (**c**) left duplicated ureters with left VUR grade VI and right VUR grade III in an 8-week-old female.

**Figure 17:**
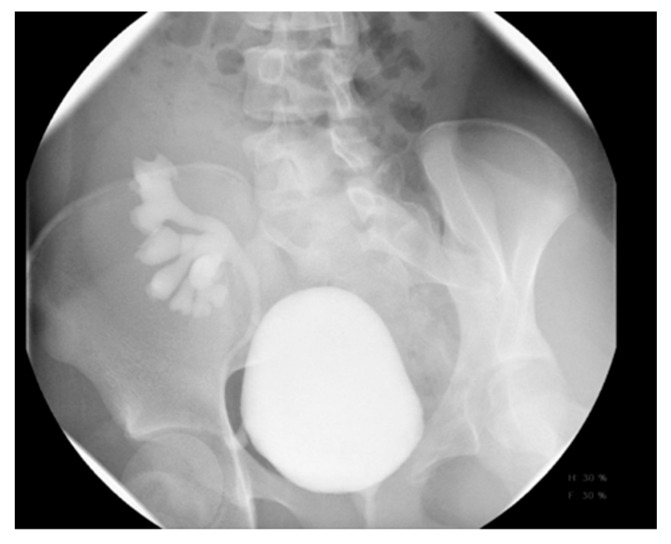
Voiding cystourethrogram showing VUR to a transplanted kidney in an 18-year-old female.

VCUG is the gold standard for diagnosing VUR, classified into five grades by the International Reflux Study in Children. High-grade VUR (grades IV and V) may show intrarenal reflux (Figure 16 and Figure 17) [36]. Renal US has low sensitivity and specificity for diagnosing VUR [63,64]. RNC and CEVUS are effective alternatives to VCUG, offering less radiation exposure.

## 17. Cystic Kidney Disease

**Figure 18:**
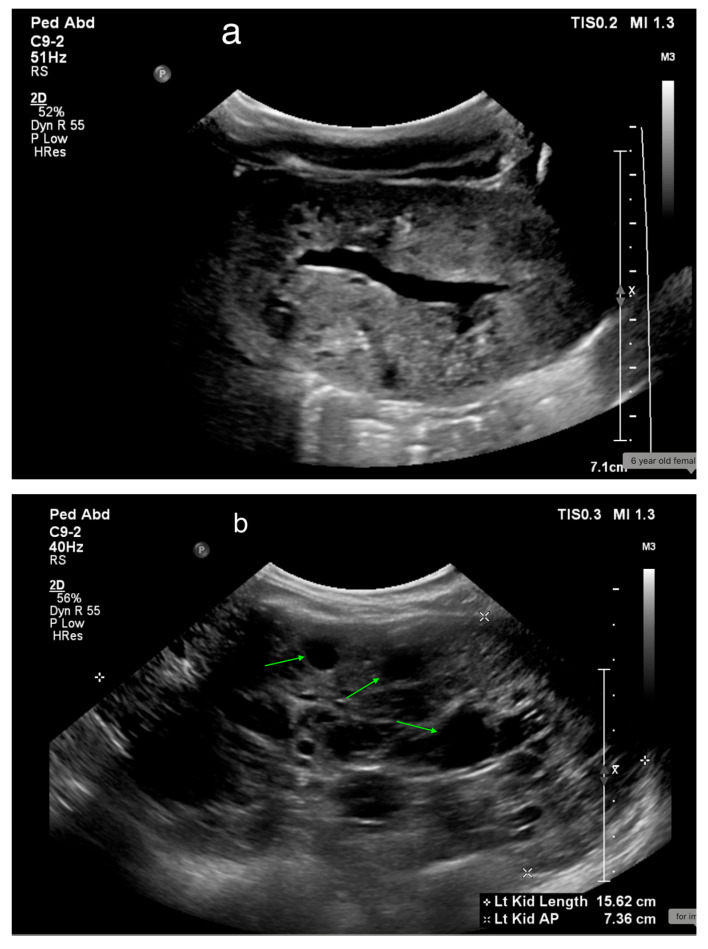
(**a**) ARPKD in a 5-day-old male identified by enlarged, echogenic kidneys with poor corticomedullary differentiation, without visible renal cysts. (**b**) A 6-year-old female with ARPKD, presenting with significantly enlarged kidneys and macrocysts (highlighted by green arrows).

Autosomal recessive polycystic kidney disease (ARPKD) presents with a broad clinical spectrum, from severe perinatal forms to minimal renal disease with dominant hepatic fibrosis. Ultrasound (US) is the initial diagnostic tool, typically showing enlarged, echogenic kidneys with poor corticomedullary differentiation. Microcysts are not visible on US, but macrocysts may be seen in older children (Figure 18) [65,66,67].

**Figure 19:**
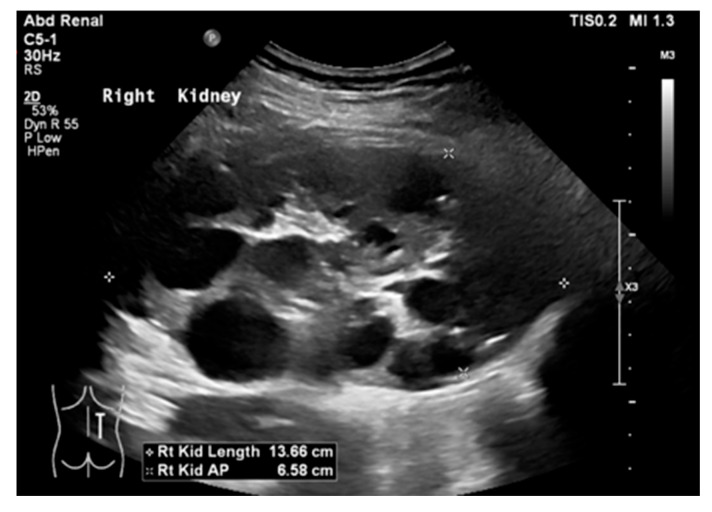
Autosomal dominant polycystic kidney disease in a 16-year-old female.

Autosomal dominant polycystic kidney disease (ADPKD) is marked by multiple cysts in both kidneys. US, MRI, and CT are useful for detecting renal cysts and complications (Figure 19) [68]. Cysts may also be present in the liver, spleen, and pancreas. The number and size of renal cysts in children increase with age [69]. Renal failure due to ADPKD is rare in children [70,71]. In adults, MRI is used to assess renal size and predict functional decline [68].

## 18. Tuberous Sclerosis

**Figure 20:**
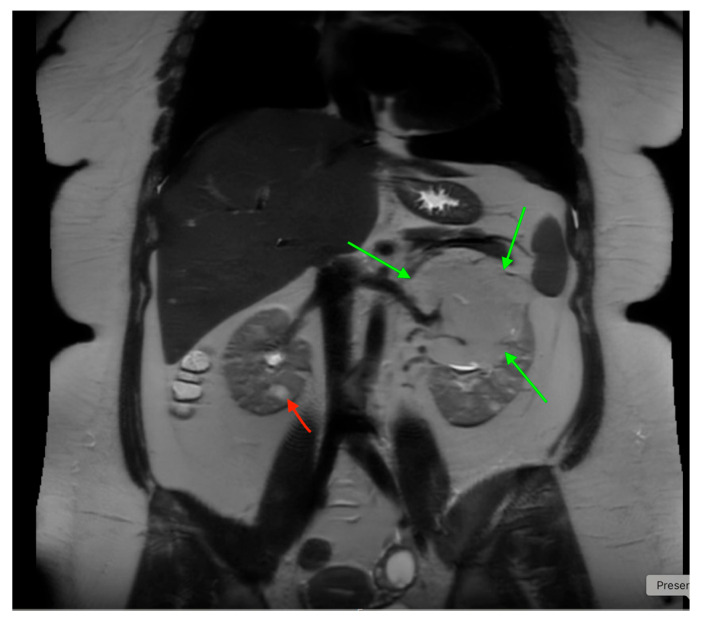
An abdominal MRI of an 18-year-old female with tuberous sclerosis reveals a large angiomyolipoma in the left kidney (marked by green arrows) and a smaller angiomyolipoma in the right kidney (marked by a red arrow).

**Figure 21:**
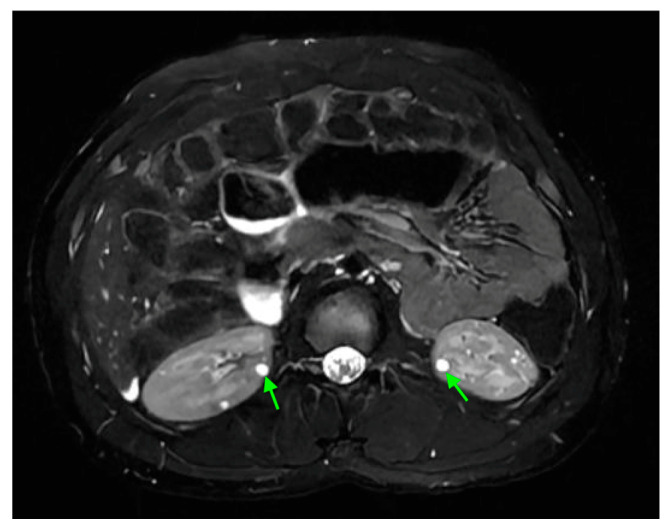
Abdominal MRI of a 16-year-old female with tuberous sclerosis, showing bilateral renal cysts (indicated by green arrows).

US, CT, and MRI are helpful in detecting angiomyolipomas, the most common renal lesions in tuberous sclerosis (TSC) (Figure 20). Some children with PKD1 and TSC2 mutations may develop severe cystic disease and are at risk for early renal failure (Figure 21) [72]. Children with TS should have a renal ultrasound at diagnosis and follow-up every 1–3 years [73]. Renal malignancies, such as malignant angiomyolipoma and renal cell carcinoma, are concerns in TSC and can occur in children [74,75]. Patients with rapidly changing lesions need more frequent imaging, and CT or MRI may be required to screen for malignant changes in severe kidney disease.

## 19. Kidney Failure

US is the initial imaging modality for patients with unexplained renal failure. It helps assess renal morphology, size, echogenicity, hydronephrosis, and cystic disease [2]. In chronic kidney disease (CKD), US may show small, echogenic kidneys with a thin cortex, aiding in the differentiation from acute kidney injury (AKI). US is also useful for detecting congenital anomalies of the kidneys and urinary tract (CAKUT), a leading cause of end-stage kidney disease in children. If CKD is due to polycystic kidney disease, the kidneys may appear enlarged. Renal parenchymal disease is typically associated with increased echogenicity, though US results may be normal. In pre-renal AKI, kidneys appear normal on US, while glomerular disease may show echogenic and enlarged kidneys [76,77]. Generally, US findings are not highly specific for renal parenchymal disease.

## 20. Nephrolithiasis

**Figure 22:**
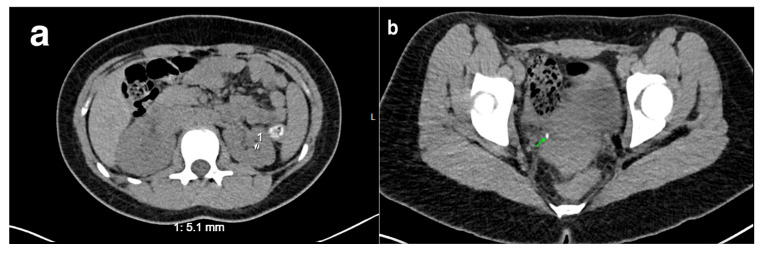
Nephrolithiasis in a 16-year-old female (indicated by number 1). (**a**) CT reveals a left intrarenal stone. (**b**) CT highlights a stone at the right ureterovesical junction (indicated by the green arrow).

US is a reliable method for detecting kidney stones as well as stones in the proximal and distal ureters. However, it often fails to detect stones in the middle portion of the ureter. Hydronephrosis caused by obstruction, though, may help confirm the diagnosis [78]. Computed tomography (CT), on the other hand, offers greater sensitivity than US, particularly for detecting renal stones and bladder calculi (Figure 22). A retrospective study conducted by JEFFREY S. et al. reviewed the charts of 75 children aged 0 to 18 years with urolithiasis. Of these, 54 patients (72%) exhibited symptoms of urolithiasis, such as flank pain and gross hematuria. CT scans were highly accurate in detecting stones, with accuracy rates ranging from 96% to 100% for both symptomatic and asymptomatic patients. In contrast, ultrasound showed more variability in accuracy, particularly in symptomatic patients (ranging from 33% to 100%), and missed urolithiasis in 41% of symptomatic cases, compared to just 5% with CT. CT also demonstrated consistent accuracy regardless of the location of the stones, while ultrasound was less effective at detecting stones in the ureter (38%) compared to the kidney (90%) [25].

## 21. Trauma to the Urinary Tract

**Figure 23:**
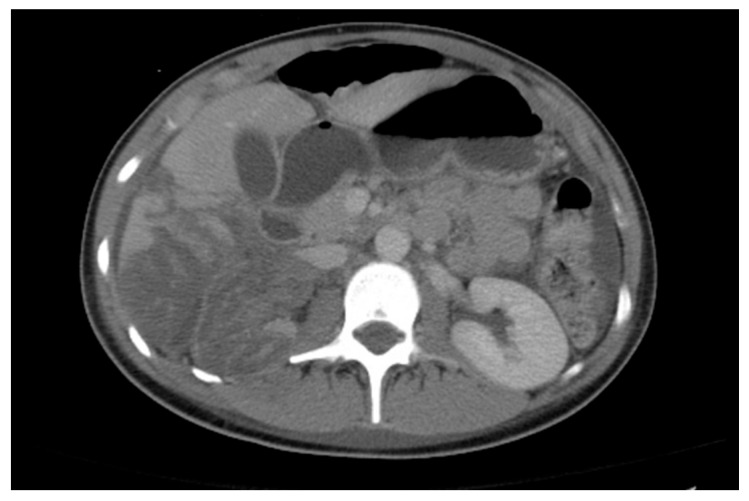
Largely infarcted right kidney in an 18-year-old female involved in a motor vehicle collision.

US may be used to detect large lacerations, urinomas, and hematomas in patients with suspected renal trauma. US with Doppler can be performed to identify significant vascular injuries; however, CT with contrast is the best modality for evaluating a patient with renal trauma (Figure 23) [79]. CT angiography provides detailed information about the renal arteries and veins. VCUG can help detect urethral injuries.

## 22. Renal Vascular Disease

### 22.1. Renal Artery Stenosis

**Figure 24:**
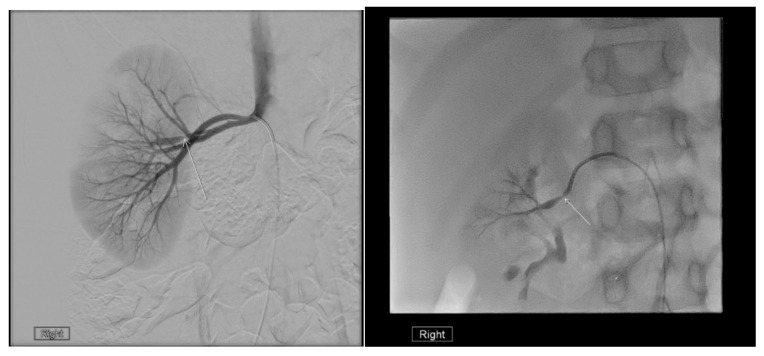
Angiography demonstrating focal stenosis (indicated by the arrows) in the segmental artery supplying the midportion of the right kidney in a 12-year-old male.

Doppler US can be used to detect renal artery stenosis, but its sensitivity is limited [80]. MRI and CT angiography are more sensitive than US and can provide high-resolution images of the renal artery. However, they do not visualize distal branch artery lesions. Conventional angiography remains necessary to evaluate renal artery stenosis if it is clinically suspected (Figure 24) [80,81].

### 22.2. Renal Vein Thrombosis

**Figure 25:**
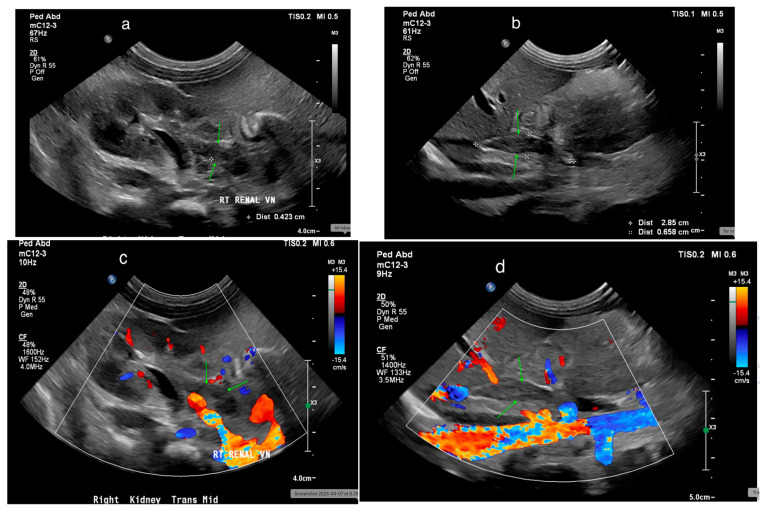
(**a**) Thrombosis in the renal vein (green arrows) in a 7-day-old male. (**b**) Thrombosis in the inferior vena cava (IVC) (green arrows) in a 7-day-old male. (**c**) Doppler ultrasound (US) shows minimal flow around the thrombus in the right renal vein (green arrows). (**d**) Doppler US shows no flow around the thrombus in the IVC (green arrows).

US effectively detects renal vein thrombosis (RVT), with its appearance varying over time. In the first few days, echogenic streaks may appear due to thrombi in interlobular and interlobar veins [82]. By the first week, kidney enlargement and an echogenic cortex may develop. Later, corticomedullary differentiation loss becomes prominent, and thrombus calcification may be seen [83]. US can also detect thrombi in the renal vein and inferior vena cava (Figure 25a,b). Doppler US may show an elevated resistance index (RI) and absent renal venous flow (Figure 25c,d) [82,83].

### 22.3. Nutcracker Syndrome

**Figure 26:**
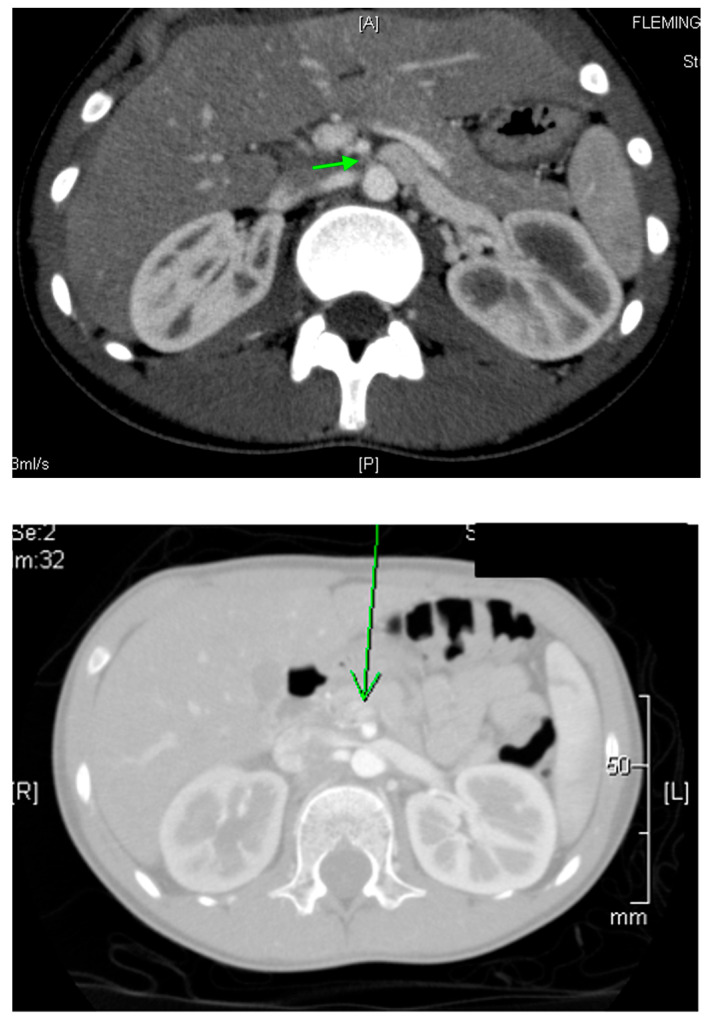
CT with angiography shows the compression of the left renal vein between the aorta and the proximal superior mesenteric artery as indicated by green arrows (Nutcracker Syndrome).

Nutcracker syndrome refers to the compression of the left renal vein between the aorta and the proximal superior mesenteric artery. Doppler US, MRI, and CT scanning are used to confirm the diagnosis of Nutcracker syndrome (Figure 26) [84].

## 23. Urinary Tract Tumors

US is useful for initial tumor evaluation, but CT or MRI is needed to assess extent and metastases. It is particularly sensitive for detecting bladder tumors in children [85]. Both US and CT aid in evaluating and monitoring Wilms tumor [86,87].

**Figure 27:**
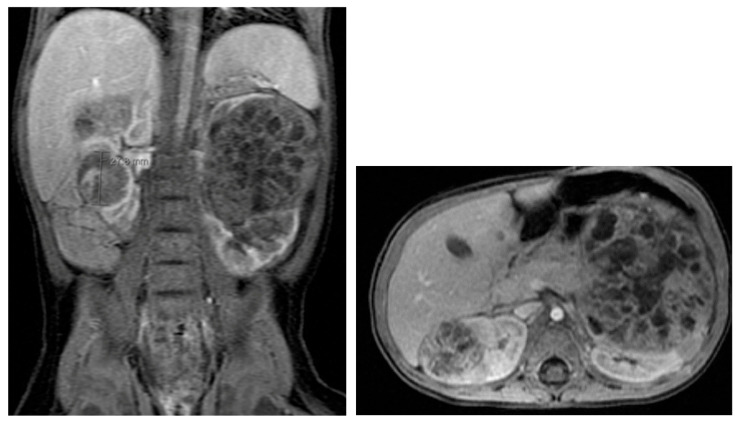
MRI shows bilateral nephroblastomatosis with bilateral nephroblastoma in a 2-year-old female.

For Wilms tumor staging, US should assess the abdomen and pelvis, while X-rays and CT are used for the chest, abdomen, and pelvis. MRI provides a comprehensive abdominal view and is effective for evaluation (Figure 27) [86]. US screens for tumor recurrence, though CT and MRI may be required to detect nephrogenic rests, the precursors of Wilms tumor [86]. US is preferred for screening high-risk children, such as those with Beckwith–Wiedemann syndrome, hemihypertrophy, or Denys–Drash syndrome [88].

## 24. Conclusions

This review highlights the essential role of diagnostic imaging in evaluating urinary tract disorders. US remains the first-line modality due to its safety and cost-effectiveness, while CT is superior for detecting renal calculi and trauma but raises concerns about radiation exposure. MRI provides excellent soft-tissue contrast without radiation but is expensive and often requires sedation. Nuclear medicine techniques offer functional assessments crucial for specific conditions, and VCUG remains indispensable for diagnosing vesicoureteral reflux. Future advancements in imaging, including AI-assisted diagnostics, contrast-enhanced ultrasound, and low-radiation CT protocols, have the potential to enhance diagnostic accuracy and patient safety. A tailored approach to imaging selection, based on clinical indications and patient-specific factors, remains essential for optimal patient care.

## Figures and Tables

**Table 1:** Literature Matrix for Pediatric Renal Imaging.

Study	Key Objective	Methodology/Type of Imaging	Findings/Results	Clinical Relevance
Fried JG, Morgan MA (2019) [1].	Overview of renal imaging techniques	Imaging techniques for kidneys (US, CT, MRI)	Summarizes core imaging methods	Comprehensive review of renal imaging applications
Viteri B, et al. (2020) [2].	Renal imaging in pediatric patients	Pediatric-specific renal imaging (US, MRI)	Discusses renal imaging modalities in children	Highlights importance of specialized pediatric imaging
Maliborski A, et al. (2018) [3].	Diagnostic imaging in pediatric kidney diseases	Multiple methods (US, MRI, CT, Nuclear Medicine)	Differentiates kidney diseases in children	Useful for diagnosing congenital/acquired pediatric kidney conditions
Kher KK, et al. (2017) [5].	Diagnostic imaging of the urinary tract	Pediatric nephrology imaging (US, VCUG, MRI)	Reviews urinary tract disease imaging in children	Key resource for pediatric nephrology professionals
O’Neill WC (2014) [6].	Applications of ultrasound in nephrology	Ultrasound for kidney disease	US essential for evaluating kidney function	Practical guide for nephrologists/radiologists
Riccabona M (2014) [7].	Pediatric ultrasonography techniques	Pediatric ultrasound	Discusses techniques, principles, and applications	Essential for clinicians using US in pediatric care
Chiara A, et al. (1989) [9].	Measurement of kidney length in infants	US for kidney length measurement	Provides kidney length norms for full/preterm infants	Crucial for diagnosing developmental kidney anomalies
Rosenbaum DM, et al. (1984) [10].	Normal renal size assessment in children	US	Examines normal kidney length in healthy children	Important for establishing normal kidney size
Palmer JS, et al. (2005) [11].	Diagnosis of pediatric urolithiasis	US and CT	Reviews role of imaging in kidney stone diagnosis	Guides imaging decisions for suspected pediatric urolithiasis
Nguyen HT, et al. (2014) [12].	Classification of urinary tract dilation	US for prenatal/postnatal diagnosis	Establishes UTD classification consensus	Key for assessing urinary tract dilation in children
Darge K, et al. (2011) [13].	MRI in pediatric nephro-urology	MRI for pediatric renal/urinary imaging	MRI offers superior contrast without radiation	Supports increased MRI use in pediatric imaging
Jequier S, Jequier JC (1989) [14].	Assess accuracy of VCUG in detecting vesicoureteral reflux	VCUG for vesicoureteral reflux detection	Evaluates reliability of VCUG for reflux diagnosis	Helps refine diagnostic protocols for pediatric urinary reflux
Piepsz A, et al. (2006) [15]	Radionuclide imaging in pediatric nephrology	DMSA and MAG3 renal scans	Highlights nuclear medicine’s diagnostic value	Critical for functional renal damage assessment

**Table 2:** Normal Kidney Length by Age.

Age	Mean Length (cm)	Range (±2 SD in cm)
Term newborn	4.48	3.86–5.10
2 months	5.28	3.96–6.60
6 months	6.15	4.81–7.49
1.5 years	6.65	5.57–7.73
2.5 years	7.36	6.28–8.44
3.5 years	7.36	6.18–8.54
4.5 years	7.78	6.87–8.87
5.5 years	8.09	7.01–9.17
6.5 years	7.83	6.39–9.27
7.5 years	8.33	7.31–9.35
8.5 years	8.90	7.14–10.66
9.5 years	9.20	7.40–11.00
10.5 years	9.17	7.53–10.81
11.5 years	9.60	8.32–10.88
12.5 years	10.42	8.68–12.16
13.5 years	9.79	8.29–11.29
14.5 years	10.05	8.81–11.29
15.5 years	10.93	9.41–12.45
16.5 years	10.04	8.32–11.76
17.5 years	10.53	9.95–11.11
18.5 years	10.81	8.55–13.07

Adapted from Rosenbaum et al. [10].

**Table 3:** Society for Fetal Urology Grading System for Hydronephrosis.

Grade	Ultrasound Description
0	No hydronephrosis
1	Only the renal pelvis is visible
2	Renal pelvis and a few calyces visible
3	Virtually all calyces visible
4	Similar to grade 3, but with parenchymal thinning

## Data Availability

No datasets were generated or analyzed in this manuscript.
